# Unveiling the social and environmental benefits of digital transformation in corporations

**DOI:** 10.1371/journal.pone.0320064

**Published:** 2025-03-14

**Authors:** Biru Cao, Tianli Wang, Ang Li, Yujie Shang, Jinghao Zhu

**Affiliations:** 1 School of Public Finance and Taxation, Capital University of Economics and Business, Beijing, China; 2 School of Government, Central University of Finance and Economics, Beijing, China; 3 Dongwu Business School, Soochow University, Jiangsu, China; University of Doha for Science and Technology, QATAR

## Abstract

In the context of the “dual-carbon” goal and the digital economy, exploring the impact of digital transformation on enterprises’ social and environmental responsibility is a key issue for achieving sustainable enterprise development and promoting high-quality economic development. This study empirically examines the impact of digital transformation on enterprises’ social and environmental responsibility and its mechanism. We achieved this through using instrumental variables and DID (Differences-in-Differences)models and selecting the data of Chinese A-share listed enterprises from 2008 to 2021. The study concludes that an enterprise’s digital transformation positively contributes to the fulfilment of corporate social responsibility. However, the digital transformation of the enterprise has a dampening effect on the fulfilment of the enterprise’s environmental responsibility. Additionally, this effect holds after a series of robustness tests. Further investigation shows that financial constraints have a positive moderating effect on enterprise digital transformation and corporate social responsibility(CSR) and a negative moderating effect on enterprise environmental responsibility. In addition, we found that the impact of enterprise digital transformation on CSR and environmental responsibility varies by firm type. The above studies provide valuable practical experiences for enterprises to achieve green and low-carbon development, reduce environmental pollution, and realize high-quality economic development as well as insight for enterprises and policy implementers.

## 1. Introduction

On September 22, 2020, China formally announced its “*Dual Carbon*” goals during the 75th United Nations General Assembly, setting the targets of *peaking carbon dioxide emissions* by 2030 and *carbon neutrality* by 2060. This ambitious initiative is expected to expedite the domestic adoption of green and low-carbon industries, fundamentally reshaping businesses’ development philosophy. This signifies that companies must not focus solely on enhancing product quality and operational efficiency for economic gains; they must also underscore the importance of assuming social and environmental responsibilities, aligning with policy requirements and the prevailing trends of the era. However, social and environmental responsibility as a public good has a positive externality. If an enterprise’s investment in social and environmental responsibility does not necessarily bring economic benefits, it will inevitably result in irreconcilable contradictions between its commitment to social and environmental responsibility and its goal of profit maximization. This will lead to insufficient intrinsic motivation for the enterprise to take the initiative to undertake social and environmental responsibility [1]. Therefore, under the requirements of China’s high-quality economic development and the “dual-carbon” goal, exploring the influencing factors of corporate social responsibility (CSR) and environmental responsibility and elaborating on their functioning mechanisms has become a key issue to be solved in the process of China’s green and low-carbon development as well as the greening of enterprises.

The integration of digital innovations across various economic and social sectors is a potent catalyst for the “*Dual Carbon”* strategy and the advancement of green technologies. Moreover, it plays a pivotal role in fostering a sustainable green economic framework and reconfiguring the organization and production of firms [2,3]. The digital economy has emerged as a pivotal mode of global economic development, with blockchain serving as the foundation of the future digital landscape. This has propelled a new wave of global industrial chain restructuring, influenced novel industry layouts, and driven consumption and investment growth. Moreover, it effectively bolsters total factor productivity in traditional physical industries [4,5], functioning as a transformative engine for economic and societal change.

The profound integration of digital technologies within firms significantly affects traditional firm management, development strategies, and productivity growth. Empowering non-economic performance and uncovering the intrinsic mechanisms that enable firms to shoulder social and environmental responsibilities have become increasingly central concerns in academic research [6,7]. Through digital transformation, companies enhance the flexibility and coordination of resources. This has led to improved efficiency in human resource allocation and innovation resource integration and triggered changes in the working process, structure of production factor distribution, and even business mode. In turn, this reduces the negative impact of resource allocation transaction costs on firms’ economic benefits [8,9]. Accenture’s recent report titled “2022 China Enterprise Digital Transformation Index” reveals that 17% of Chinese firms successfully underwent digital transformation in 2022, a slight increase from 16% in 2021. Additionally, nearly 60% of the surveyed executives expressed their intent to boost their investments in digital transformation over the next two years. Digital transformation has become an important strategy and inevitable choice for future development reforms and the transformation of enterprises. Therefore, this study explores the impact of digital transformation on corporate social and environmental responsibility. This is of great practical and policy significance to promote the development of the digital economy and the greening of enterprises, reduce environmental pollution, and drive high-quality economic and environmental development.

A wealth of research on digital transformation has yielded valuable insight and theoretical inspiration regarding its positive impact on firm development in terms of production efficiency, industrial technology, and strategic changes [10–12]. For example, Loebbecke and Picot argue that enterprise digital transformation can increase investment in R&D and technology, improve total factor productivity of the enterprise, and thus increase the profit of the enterprise [13]. Nasiri et al. argue that enterprise digital transformation can only contribute to the financial performance of the enterprise when it reaches a certain level [14]. Vial believes that digital strategy has become an important strategy for enterprises and continues to accumulate digital resources to enhance their agility, networking, and digital capabilities [15]. Relatively little research has been conducted on non-economic performance related to digital transformation. Few scholars have focused on the potential impact of digital transformation on the social and environmental responsibilities of enterprises. Yang et al. argue that enterprise digital transformation improves corporate social responsibility fulfilment by enhancing corporate green innovation and strengthening internal controls [16]. Fang et al. study the impact of enterprise digital transformation on corporate environmental social responsibility (CSR) from two aspects: source control and end-of-pipe management. They find that enterprise digital transformation has a significant positive impact on corporate “source control” CSR and a significant negative impact on “end-of-pipe management” CSR [17].

However, existing studies have neglected the non-economic performance aspects of enterprise digital transformation; most studies focus on the economic and internal financial performance of enterprises, and there is a relative lack of studies on non-economic performance (social or environmental responsibility). In addition, most current studies only examine the impact of the digital transformation of enterprises on non-economic performance (e.g., social or environmental responsibility) from a single dimension, and there is a paucity of articles that address both. The shortcomings of the current literature provide room for improvement. Therefore, exploring the impact of the digital transformation of enterprises and corporate social and environmental responsibility is of great theoretical and practical significance for promoting enterprises’ sustainable development and achieving high-quality economic development. Thus, this area is worthy of in-depth research.

Based on the above background, this study selects data on China’s A-share listed companies from 2008 to 2021, uses Python technology to construct enterprise digital transformation indicators, and explores the impact of enterprise digital transformation on corporate social responsibility and environmental responsibility at the theoretical and empirical levels. Compared with the existing research, the marginal contributions of this study are as follows. First, it examines the impact of enterprise digital transformation on the non-economic performance of enterprises from the dimensions of social responsibility and environmental responsibility, which expands the research on enterprise digital transformation and non-economic performance. Second, it explores the intrinsic mechanism of financing constraints in digital enterprises to drive enterprises to fulfill their social and environmental responsibilities. Finally, it explores the heterogeneous impact of enterprise digital transformation on corporate social and environmental responsibility and examines the differences in the impact of corporate social and environmental responsibility performance across different property rights, high-tech levels, and industry categories. This study enriches and expands the research on digital transformation in terms of non-economic performance. Moreover, it has practical value in promoting the implementation of carbon emission reduction responsibility, green transformation, and upgrading of enterprises in China. Finally, it is instructive for enterprises in forming the dual competitive advantages of digital transformation and social and environmental responsibility.

## 2. Theoretical analysis and research hypothesis

### 2.1 Enterprise digital transformation and CSR and environmental responsibility

In the context of the implementation of enterprise digital transformation, enterprises can use digital technology to standardize information that is less efficiently utilized in the market. This allows for improvement of the speed of their information exchange, thereby enhancing the transparency of information in the enterprise [18]. Furthermore, shareholders are able to monitor the behavior of managers in a timely and effective manner. Doing so will, to a certain extent, reduce the decision-making errors caused by external information mismatch and the “backdoor operation” behaviors generated by the opportunistic motives of managers, motivate them to pay more attention to the long-term interests of the enterprise in decision-making, improve the quality of internal control, and proactively take on social responsibility [19]. This is the main reason for the lack of a lag in social responsibility after an enterprise’s digital transformation. In addition, digital transformation can mitigate information asymmetry by reducing the cost of information exchange between enterprises and stakeholders [20] as a way to reduce its negative impact on corporate social responsibility. However, the enhancement of total factor productivity will become a fundamental impetus for enterprises to assume social responsibility. The positive nonlinear relationship between the digital economy index and provincial total factor productivity indicates that the digital economy is an innovative driver for the broad and sustainable development of total factor productivity [4]. Moreover, it suggests that enterprises will be more willing to fulfil their social mission after improving their productivity and business management level with digitalization technology to satisfy their own economic interests [21]. Furthermore, digital transformation is also driving the service-oriented transformation of enterprises, which will result in a shift in the governance philosophy of enterprises from the traditional “shareholder-centred” to the “CSR philosophy.” This will lead to an increased emphasis on product quality, brand image, and external reputation and an enhanced subjective willingness of enterprises to assume greater social responsibility.

The investment of corporate resources in environmental responsibility may prove to be a futile exercise because it may result in the expenditure of additional funds and the imposition of adverse consequences detrimental to shareholders’ interests [22]. Organizational hypocrisy theory posits that the divergent claims of stakeholder groups result in disparate perceptions of enterprise digital transformation actions. These perceptions, which may be reflected positively or negatively, may also elicit skepticism. This, in turn, may prompt investors to adopt a more conservative approach to their investments, which could have a detrimental impact on the stability of an enterprise’s share price [23]. Furthermore, the implementation of enterprise digital transformation necessitates the replacement of a multitude of obsolete equipment and facilities and the introduction of advanced digital technologies, which occupy a larger portion of the enterprise’s capital investment. This results in a shortage of funds and an adverse impact on the enterprise’s accounting performance in the current period, which may also have a detrimental effect on the stability of the enterprise’s share prices. Although the financial shortfalls and risks of share price volatility faced by enterprises may be mitigated by the increased economic benefits of digital transformation activities, enterprises do not typically demonstrate a significantly positive attitude toward environmental responsibility following digital transformation. Moreover, even when enterprises are willing to assume environmental responsibility, such behavior often exhibits a significant lag. The discrepancy between the stability of executive remuneration and the objective of corporate environmental responsibility also contributes to executives’ reluctance to invest in environmental protection at the expense of the firm’s current interests, in the expectation of inherently uncertain long-term future benefits. Performance-based payout mechanisms may reduce managers’ incentives to invest in the long term [24]. As a result, they may pursue short-term accounting performance, thereby abandoning the future development strategy of assuming environmental responsibility. Although enterprise digital transformation improves the efficiency of internal monitoring by enterprise stakeholders through the improvement of internal information transparency, which, to a certain extent, avoids the opportunistic tendency of executives and solves part of the principal-agent problem, the assumption of external environmental responsibility will to a certain extent infringe on the economic interests of the enterprise. The enterprise in the present and in a longer period in the future is uncertain about the amount and time of economic rewards brought about by this part of the cost investment. This is because of the self-interested tendencies of individual executives. Ultimately, this creates unfavorable reciprocal value mechanisms for the sustainability of the assumption of corporate environmental responsibility. The amount and time of economic rewards brought about by this part of the cost investment for the enterprise are full of uncertainty owing to the self-interested tendencies of individual executives. This ultimately results in a reciprocal value mechanism that is unfavorable to the enterprise’s external stakeholders, which has a negative impact on the sustainability of the enterprise’s environmental responsibility.

Based on the above discussion, the following hypotheses are proposed in this paper:

H1a: Enterprise digital transformation can significantly improve corporate social responsibility performance.

H1b: Enterprise digital transformation can significantly reduce an organization’s environmental responsibility performance.

### 2.2 Enterprise digital transformation, financing constraints, and enterprise social and environmental responsibility

Financing constraint can be defined as a situation in which an enterprise is unable to invest because of market mechanism imperfections. This results in a significant gap between the cost of funds raised internally and those raised externally. Therefore, the cost of external financing is higher, which may lead the enterprise to rely more on internal funds, thus forming an external financing constraint [25]. Financial constraints imply a reduced availability of external financing and a lower level of stakeholder support for the firm. When firms face operational difficulties, they are more willing to seek strategic change and have a greater tendency to engage in riskier innovative activities to gain stakeholder support [26]. On one hand, when enterprises are subject to stronger financing constraints, they are more inclined to take on social responsibility to make their stakeholders more aware of their internal information and increase investment opportunities, thus reducing the difficulty of financing their enterprises. Being socially responsible is a human-oriented behavior, and the process of enterprise digital transformation facilitates the fulfilment of corporate social responsibility. This is because the digital transformation of enterprises can help reduce the cost of information transmission between stakeholders. Furthermore, it can improve the transparency of information of enterprise stakeholders as a way to build positive expectations of stakeholders about the future development of the enterprise, seek cooperation with enterprise stakeholders [27]. Additionally, it can attract new investment to alleviate the current shortage of funds for the enterprise [28]. Therefore, the stronger the financing constraints enterprises face are, the stronger the positive impact of enterprise digital transformation on CSR. On the other hand, the implementation of enterprise digital transformation requires the replacement of a large amount of obsolete equipment and facilities and the introduction of advanced digital technologies, which will occupy more of the enterprise’s capital investment, putting the enterprise in a shortfall of funds. At the same time, the disclosure of strategic decisions on digital transformation may also entail the risk of share price volatility and thus weaken the propensity of enterprises to take further risks when the financing constraints they face are higher. Because taking on environmental responsibility requires more financial support, enterprises often do not choose to make new investments in taking on environmental responsibility after digital transformation; the higher the financing constraints faced by the enterprise are, the weaker the positive impact of digital transformation on environmental responsibility performance.

Based on the above discussion, the following hypotheses are proposed in this paper:

H2a: Financing constraints produce a positive moderating effect between enterprises’ digital transformation and corporate social responsibility performance.

H2b: Financing constraints have a negative moderating effect on enterprises’ digital transformation and corporate environmental responsibility performance.

## 3. Research design

### 3.1 Data and variables

The data used in this study are from the China Stock Market and Accounting Research Database (CSMAR), which compiles indicators disclosed in firms’ CSR reports covering various aspects of social and environmental responsibilities and the word frequency of digital transformation. The CSMAR database is an accurate research-oriented database in the fields of economy and finance, developed in combination with China’s actual national conditions. As the CSMAR is one of the highest-quality databases for financial and accounting research in China, our empirical results are accurate and reliable.

We construct our outcome variables using firms’ CSR report information. In the original data, if a firm did not disclose a specific responsibility item, the corresponding variable was defined as 0. If a firm qualitatively disclosed an item, the variable was defined as 1. If a firm quantitatively disclosed an item, the variable was defined as 2. Because both qualitative and quantitative disclosures represent firms’ performance in fulfilling social responsibilities, we treat both types of disclosure as 1 to better reflect the firm’s social responsibility fulfillment status. The explanatory variables are defined as follows. First, we identify the frequency of digital transformation using five related terms: big data, cloud computing, blockchain, digital technology application, and artificial intelligence. These words were then summed to represent the degree of transformation of a firm. Specifically, if a firm disclosed any of these terms once in its annual report for a particular year, we added 1 to the digital transformation frequency variable. If the firm did not disclose any of these terms in its annual report for that year, the digital transformation frequency variable was defined as 0. In the reduced-form DID approach, we defined a new dummy variable that reflects the digital transformation stage. If a firm discloses relevant terms related to digital transformation in a particular year and onward, we assigned a value of 1 to this indicator; otherwise, we assigned a value of 0.

In our empirical model, we controlled for other variables, such as the firm’s price-to-book ratio, natural logarithm of firm age, natural logarithm of total assets, and debt ratio. We conducted a heterogeneity analysis based on firm characteristics, including whether the firm is a state-owned, high-tech, manufacturing, or heavily polluting enterprise. Furthermore, we explored the possible mechanism between a firm’s digital transformation and its fulfillment of social and environmental responsibilities using the KZ index variable, which measures financial constraints. The research period in our study spanned from 2008 to 2021. The descriptive statistics and variable descriptions in Table 1 and Table 2. It is worth noting that although we excluded data for firms with missing information on social and environmental responsibilities, most missing values were due to significant deficiencies in the responsibility reports or the lack of credibility in those reports. For firms without relevant information disclosed, we assigned a value of 0 to the corresponding variables instead of treating them as missing values. This approach minimizes the potential biases in variable selection.

**Table 1:** Variable definitions.

Variable	Description
Digital_transfer_dummy	Measuring whether the firm implemented digital transformation in a given year; a value of 1 is assigned if the company disclosed related terms such as big data, cloud computing, blockchain, digital technology application, and artificial intelligence for that year and subsequent years, otherwise, a value of 0 is assigned.
Digital_transfer	Measuring whether the firm implemented digital transformation in a given year; a count of 1 is added to the indicator if the company disclosed related terms such as big data, cloud computing, blockchain, digital technology application, and artificial intelligence once in its annual report for that year.
SystemConstruction	Disclosure of social responsibility system establishment and improvement measures (Disclosure = 1, Not disclosed = 0)
SolidWasteDispUtil_d	Solid waste utilization and disposal status (Disclosure = 1, Not disclosed = 0)
PublicRelations	Disclosure of public relations and social welfare initiatives (Disclosure = 1, Not disclosed = 0)
CustomerProtection	Disclosure of customer and consumer rights protection (Disclosure = 1, Not disclosed = 0)
ShareholdersProtection	Disclosure of shareholder rights protection (Disclosure = 1, Not disclosed = 0)
StaffProtection	Disclosure of employee rights protection (Disclosure = 1, Not disclosed = 0)
SootDustRed_d	Dust and smoke control status (Disclosure = 1, Not disclosed = 0)
WasteGasEmissRed_d	Emission reduction control of exhaust gas (Disclosure = 1, Not disclosed = 0)
WasteWaterEmissRed_d	Emission reduction control of wastewater (Disclosure = 1, Not disclosed = 0)
lnasset	Firm’s total assets (logarithmic value)
lnage	Firm’s age at establishment (logarithmic value)
PB	Firm’s price-to-book ratio disclosed in the current year’s annual report
largest shareholder ratio	Percentage of shares held by the largest shareholder disclosed in the current year’s annual report (%)
Debt to asset	Debt ratio
KZ index	KZ index reflecting financial constraints
hightech	Whether the firm is a high-tech enterprise
ownership	Whether the firm is a state-owned enterprise (State-owned = 1, Non-state-owned = 0)
madeind	Whether the firm is a manufacturing enterprise (Yes = 1, No = 0)
serviceind	Whether the firm is a service enterprise (Yes = 1, No = 0)
KeyPollMonunit	Whether the firm is a heavily-polluting enterprise (Yes = 1, No = 0)

**Table 2:** Descriptive statistics.

Variable	N	Mean	SD
Digital_transfer_dummy	36507	0.449	0.497
Digital_transfer	36507	9.161	26.468
SystemConstruction	36507	0.085	0.279
SolidWasteDispUtil_d	36507	0.247	0.431
PublicRelations	36507	0.653	0.476
CustomerProtection	36507	0.537	0.499
ShareholdersProtection	36507	0.593	0.491
StaffProtection	36507	0.661	0.473
SootDustRed_d	36507	0.168	0.374
WasteGasEmissRed_d	36507	0.302	0.459
WasteWaterEmissRed_d	36507	0.305	0.461
lnasset	36507	22.15	1.503
lnage	36507	2.835	0.378
PB	36507	4.774	26.89
largest shareholder ratio	36507	34.61	15.19
Debt to asset	36507	0.436	0.218
KZ index	31867	1.215	2.491
hightech	36507	0.306	0.461
ownership	36507	0.379	0.485
madeind	36507	0.632	0.482
serviceind	36507	0.194	0.396
KeyPollMonunit	32456	0.126	0.332

### 3.2 Empirical methodology

We implemented a fixed-effects model, instrumental variable two-stage regression, and reduced-form difference-in-differences (DID) approach to assess the relationship between digital transformation and corporate social and environmental responsibilities. Our fixed effects model is shown in formula (1), where the subscripts represent the firm (i), time (t), city (c), and province (p). We controlled for firm-specific fixed effects and time fixed effects in the main regression model and conducted robustness tests by controlling for city and province fixed effects to ensure the robustness of our results. Additionally, we include the variables mentioned in the descriptive statistics, such as firms’ price-to-book ratio, logarithm of firm age, logarithm of total assets, debt ratio, and percentage of shares held by the largest shareholder, as control variables to capture firm ownership, market characteristics, and firm-specific characteristics. These variables may influence the disclosure of environmental and social responsibilities, and their inclusion helps address potential omitted variable issues. Our main independent variable was the degree of a firm’s digital transformation. In the fixed effects model, we used the logarithm of the total frequency count of the five terms related to digital transformation as the main variable, while the dependent variable was a dummy variable representing the information disclosed by the firm’s corporate social responsibility (CSR) report regarding environmental and social responsibility fulfillment.


$$Y_{i,t,c,p}=\beta_{0}+\beta_{1}Transfer_{i,t}+\beta_{2}X_{i,t,c,p}+\lambda_{i,c,p}+\mu_{t}+\varepsilon_{i,g,c,p}$$ (1)

In our instrumental variable and two-stage regression models, as shown in formulas (2) and (3), we use the average digital transformation frequency count in the firm’s industry, excluding the firm itself, as the instrument for the firm’s digital transformation frequency count. We followed the 2012 China Securities Regulatory Commission Industry Classification Guidelines as our industry classification, with the 13 first-level industries specified in the guidelines as industry classification indicators to construct our instrument. Next, we regressed the firm’s digital transformation and other control variables on the total frequency count of the digital transformation terms in the firm’s industry, where i represents the average value, excluding the specific firm (i). After obtaining the regression estimates, we incorporated them into the second-stage regression, controlling for fixed effects and other variables, to examine the impact of our core independent variable on the dependent variable.

We selected the average digital transformation level of the industry, excluding the focal company, as an instrumental variable. This choice was informed by several critical considerations. First, the instrumental variable is highly correlated with the target company’s degree of digital transformation, as firms’ digitalization decisions are generally influenced by industry trends. Second, this instrumental variable satisfies the condition of exogeneity because it reflects the average behavior of other companies within the industry, unrelated to any specific conditions of the focal company, thereby mitigating potential endogeneity issues. Furthermore, employing the first-level industry classification based on the 2012 CSRC (China Securities Regulatory Commission) Industry Classification Guidelines ensures precise and authoritative industry segmentation, enhancing the representativeness and accuracy of the industry-average data. This precise classification method strengthens the explanatory power and predictive accuracy of the model, effectively addressing the potential endogeneity problems encountered in traditional regression analysis. These factors collectively affirm the rationality and applicability of the chosen instrumental variable, making the findings of this study reliable and persuasive.

First-stage estimation is as shown below:


$$Average\hat{T}ransfer_{-i,t}=\gamma_{0}+\gamma_{1}Transfer_{i,t}+\gamma_{2}X_{i,g,c,p}+\lambda_{i,c,p}+\mu_{t}+\varepsilon_{i,t,c,p}$$ (2)

Second-stage estimation is shown as below:


$$Y_{i,t,c,p}=\beta_{0}+\beta_{1}Average\hat{T}ransfer_{-i,t}+\beta_{2}X_{i,g,c,p}+\lambda_{i,c,p}+\mu_{t}+\varepsilon_{i,t,c,p}$$ (3)

The fixed-effects model, instrumental variables, and two-stage regression models addressed endogeneity concerns to some extent. However, potential endogeneity issues remain due to reverse causality or omitted variables. To address this, we adopted the reduced-form DID model, as shown in formula (4), using the core variables representing social and environmental responsibilities as the dependent variables and employing a specific identification strategy. In this strategy, we defined a firm as having implemented digital transformation for a particular year and subsequent years if it disclosed related terms such as big data, cloud computing, blockchain, digital technology applications, and artificial intelligence for those years. We assigned a value of 1 to the indicator for implementing digital transformation in that year and subsequent years; otherwise, we assigned a value of 0. Furthermore, we defined the first year of digital transformation implementation for a firm as g = 0 and increment or decrement the corresponding years accordingly. This logic serves as an indicator of the symbolic function. If a firm is n years after the digital transformation, then g = n. Following this logic, we incorporated the symbolic function into the reduced-form DID model, including all relevant time periods in the analysis. As our data spans from 2008 to 2021, g can take any value from -14 to 14. However, the number of firms with g values less than -10 and greater than 10 was small, accounting for only 6% of the total sample. To avoid the undue influence of these extreme values on the estimation results, we truncated the samples with g values less than -10 and greater than 10 to -10 and 10, thereby constructing the core identification item for the reduced-form DID model. We control for time fixed effects, firm-specific fixed effects, and all other control variables mentioned earlier in the model to obtain the results of the reduced-form DID model. This identification strategy aligns with that of Chen et al. [29].


$$Y_{i,t,c,p}=\beta_{0}+\overset{10}{\underset{\alpha =-10}{\sum }}\beta_{1,\alpha }I\left(g=\alpha \right)+\beta_{2}X_{i,t,c,p}+\lambda_{i,c,p}+\mu_{t}+\varepsilon_{i,t,c,p}$$ (4)

## 4. Empirical results

### 4.1 Baseline regression

To provide a more comprehensive understanding of the controlled independent variables and to demonstrate their relationships without confounding the main regression results with inter-variable correlations, we examined the correlations among all variables and present the correlation coefficient matrix in Table 3. Notably, the highest correlation coefficient is observed between the debt ratio and the logarithm of total assets, which can be attributed to both variables using total assets as a common factor in their definitions. In summary, all variables exhibited statistically significant correlations at the 5% significance level. However, excepting the correlation between the logarithm of total assets and the debt ratio, the absolute values of the correlation coefficients were all below 0.5, suggesting that the inter-variable correlations among the controlled variables did not significantly impact the robustness of our main regression results.

**Table 3:** Correlation analysis.

Variable	lnasset	lnage	PB	largest shareholder ratio	Debt to asset
lnasset	1				
lnage	0.179***	1			
PB	-0.127***	0.013**	1		
largest shareholder ratio	0.170***	-0.129***	-0.041***	1	
Debt to asset	0.504***	0.175***	0.063***	0.035***	1

Note: The asterisks * , **, and *** indicate statistically significant at the 10%, 5%, and 1% levels.

According to the Hausman test results presented in Table 4, the findings support the use of fixed effects regression models for all variables. Specifically, the chi2 statistics from the Hausman tests were significant, with p-values less than 0.01. These results indicated that fixed-effects models were more appropriate than random-effects models. Therefore, we utilized fixed-effects regression models to analyze the impact of digital transformation on corporate social and environmental responsibilities.

**Table 4:** Hausman test results.

Hausman test	Test results	conclusions
CustomerProtection	chi2(6) = 781.13, Prob > F = 0.000	fixed-effects regression
SystemConstruction	chi2(6) = 53.97, Prob > F = 0.000	fixed-effects regression
PublicRelations	chi2(6) = 2029.98, Prob > F = 0.000	fixed-effects regression
ShareholdersProtection	chi2(6) = 1256.09, Prob > F = 0.000	fixed-effects regression
StaffProtection	chi2(6) = 1767.81, Prob > F = 0.000	fixed-effects regression
SootDustRed_d	chi2(6) = 390.27, Prob > F = 0.000	fixed-effects regression
WasteGasEmissRed_d	chi2(6) = 635.35, Prob > F = 0.000	fixed-effects regression
WasteWaterEmissRed_d	chi2(6) = 635.45, Prob > F = 0.000	fixed-effects regression
SolidWasteDispUtil_d	chi2(6) = 354.04, Prob > F = 0.000	fixed-effects regression

The results of our fixed-effects regression are presented in Table 5. We controlled for time fixed effects, individual fixed effects, and the five control variables presented earlier. We then conducted a regression using the frequency of digital transformation as the independent variable and our major dependent variable as the outcome. The coefficient estimates are summarized in Table 5. Overall, we found that the frequency of digital transformation has a positive effect on a firm’s fulfillment of social responsibilities and a negative effect on its fulfillment of environmental responsibilities. Specifically, for every 1% increase in the frequency of digital transformation, the probability of a firm disclosing its efforts to protect customer and consumer rights increased by 1.4%, the probability of disclosing efforts to protect shareholder rights increased by 1.3%, and the probability of disclosing efforts to fulfill and protect employees’ social responsibilities increased by 0.7%.

**Table 5:** Fixed-effects regression results.

	(1)	(2)	(3)	(4)	(5)	(6)	(7)	(8)	(9)
Variable	CustomerProtection	SystemConstruction	PublicRelations	ShareholdersProtection	StaffProtection	SootDustRed_d	WasteGasEmissRed_d	WasteWaterEmissRed_d	SolidWasteDispUtil_d
Digital_transfer	0.014***	-0.001	0.004	0.013***	0.007***	-0.018***	-0.010***	-0.007**	-0.001
	(0.003)	(0.002)	(0.003)	(0.003)	(0.003)	(0.002)	(0.003)	(0.003)	(0.003)
Control variable	Yes	Yes	Yes	Yes	Yes	Yes	Yes	Yes	Yes
Firm/ Year FE	Yes	Yes	Yes	Yes	Yes	Yes	Yes	Yes	Yes
Constant	-1.295***	-0.454***	-1.194***	-1.188***	-1.052***	0.038	-0.498***	-0.401***	-0.743***
	(0.113)	(0.079)	(0.115)	(0.109)	(0.107)	(0.092)	(0.109)	(0.109)	(0.108)
Observations	36,035	36,035	36,035	36,035	36,035	36,035	36,035	36,035	36,035
R-squared	0.591	0.373	0.542	0.611	0.594	0.514	0.551	0.555	0.502

Notes: 1. Standard errors in parentheses. 2.The asterisks * , **, and *** indicate statistically significant at the 10%, 5%, and 1% levels.

Simultaneously, the probability of disclosing environmental governance responsibilities decreased. The disclosure probability for dust and smoke control decreased by 1.8%, exhaust gas emission reduction control decreased by 1%, and wastewater emission reduction control decreased by 0.7%. Except for the wastewater emission reduction control variable, which was significant at the 5% level, all other variables affected by digital transformation showed significance at the 1% level.

To further address endogeneity issues, we implemented the industry average of digital transformation frequency, excluding a firm’s own frequency, as an instrumental variable (IV). We conducted a two-stage least squares (2SLS) regression with IV. The first-stage regression results linking a firm’s digital transformation frequency to the industry average are presented in Table 6. The first column shows the first-stage estimates with no robustness adjustments. The second column presents the first-stage estimates after propensity score matching, as discussed later. The third row shows the robustness check results, which include propensity score matching and the removal of other control variables. Finally, the fourth row displays the first-stage estimates after controlling for city and province fixed effects in the model in column (2), along with the F-statistic for the first stage.

**Table 6:** First-stage estimates of IV-2SLS.

	(1)	(2)	(3)	(4)
Variable	IVdigital_trans	IVdigital_trans	IVdigital_trans	IVdigital_trans
Digital_transfer	0.143***	0.107***	0.110***	0.105***
	(0.003)	(0.004)	(0.004)	(0.004)
lnasset	0.008**	0.016***		0.019***
	(0.004)	(0.006)		(0.006)
lnage	0.036 *	-0.014		-0.044
	(0.022)	(0.033)		(0.033)
PB	-0.000***	-0.000***		-0.000***
	(0.000)	(0.000)		(0.000)
largest_shareholder_ratio	-0.000	-0.000		-0.001
	(0.000)	(0.000)		(0.000)
debt_to_asset	0.091***	0.120***		0.122***
	(0.017)	(0.022)		(0.022)
Firm/ Year FE	Yes	Yes	Yes	Yes
Observations	35,972	22,025	22,025	22,025
R-squared	0.087	0.054	0.051	0.054
F	505.5	175.4	980.9	172.6

Notes: 1.The instrumental variables estimation here refers to the industry average of digital transformation frequency, excluding a firm’s own frequency. 2.The first column shows the first-stage estimates of IV-2SLS without any robustness adjustments, the second column presents the first-stage estimates of IV-2SLS after propensity score matching as discussed later, the third row shows the robustness check results after propensity score matching, and the fourth row displays the first-stage estimates of IV-2SLS to the model in column (2). 3. All regressions control for time fixed effects, individual fixed effects, and the five control variables; 4. Standard errors in parentheses. 5.The asterisks * , **, and *** indicate statistically significant at the 10%, 5%, and 1% levels.

The results in Table 6 show that the F-statistics for the first-stage regressions are all substantially higher than the weak instrument threshold of 10 and are at least 17 times higher, demonstrating that weak instrument issues do not exist. Regarding the regression results of a firm’s frequency against the industry-average frequency, the degree of digital transformation is significant at the 1% level, and the coefficients in columns (2), (3), and (4) exhibit minimal differences, further confirming the stability of the instrumental variable.

Based on the first-stage regression results, we validated the reasonableness of using the industry-average digital transformation frequency as an IV. We then utilized the values predicted from the first-stage regression as the core variables for our second-stage estimation. The coefficient values obtained using the IV-2SLS regression are presented in Table 7, representing the cleanliness effects of digital transformation on the fulfillment of social and environmental responsibilities. From Table 7, the coefficients obtained from the IV-2SLS regression are consistent in direction and significance level compared to the baseline fixed-effects results.

**Table 7:** Second-stage estimates of IV-2SLS and fixed-effect.

	(1)	(2)	(3)	(4)	(5)	(6)	(7)	(8)	(9)
Variable	CustomerProtection	SystemConstruction	PublicRelations	ShareholdersProtection	StaffProtection	SootDustRed_d	WasteGasEmissRed_d	WasteWaterEmissRed_d	SolidWasteDispUtil_d
Digital_transfer	0.022**	-0.014 *	-0.007	0.038***	0.010	-0.117***	-0.092***	-0.092***	-0.055***
	(0.010)	(0.007)	(0.011)	(0.010)	(0.010)	(0.009)	(0.010)	(0.010)	(0.010)
Control variable	Yes	Yes	Yes	Yes	Yes	Yes	Yes	Yes	Yes
Firm/ Year FE	Yes	Yes	Yes	Yes	Yes	Yes	Yes	Yes	Yes
Observations	35,972	35,972	35,972	35,972	35,972	35,972	35,972	35,972	35,972
R-squared	0.013	0.001	0.010	0.012	0.012	-0.052	-0.024	-0.027	-0.009

Notes:1. All regressions control for time fixed effects, individual fixed effects, and the five control variables; 2. Standard errors in parentheses. 3.The asterisks * , **, and *** indicate statistically significant at the 10%, 5%, and 1% levels.

In general, the digital transformation of listed firms promotes the fulfillment of social responsibility and reduces the fulfillment of environmental responsibility. Specifically, for every 1% increase in a firm’s digital transformation frequency, the probability of disclosing efforts to protect customer and consumer rights increases by 2.2%, the probability of disclosing efforts to protect shareholder rights increases by 3.8%, and the probability of disclosing efforts to fulfill and protect employees’ social responsibilities increases by 1.0%. However, the influence of digital transformation on the fulfillment of employees’ social responsibilities became statistically insignificant. Simultaneously, the probability of disclosing environmental governance responsibilities continued to decrease, with the probability of disclosing dust and smoke control efforts decreasing by 11.7%, exhaust gas emission reduction control by 9.2%, wastewater emission reduction control by 9.2%, and solid waste utilization and disposal control decreasing by 5.5%.

### 4.2 PSM estimation

**Fig 1:**
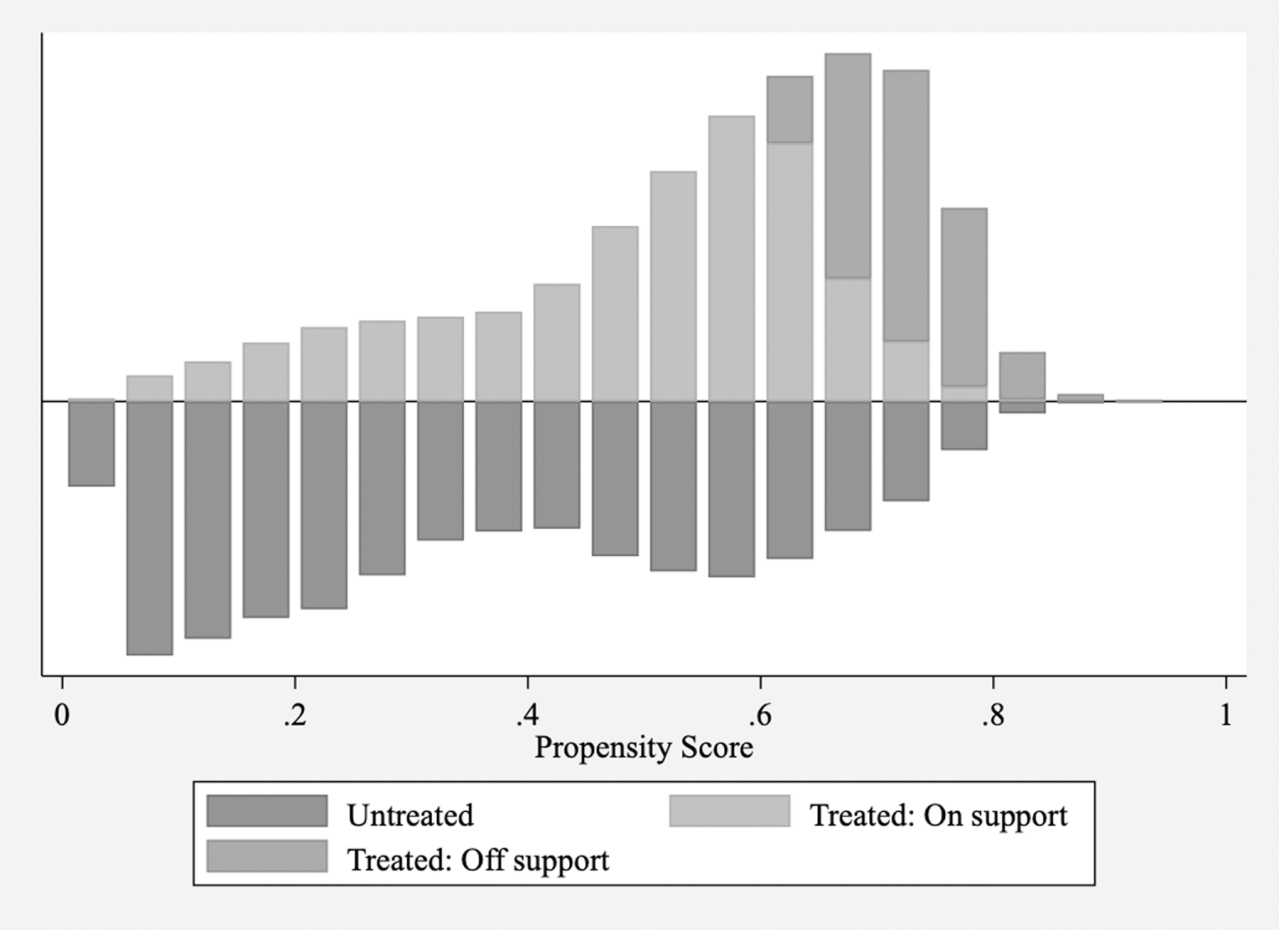
Common support process test. Notes: The color blocks from deep to light depict the common range of propensity scores for the control group, matched experimental group, and unmatched experimental group, respectively.

**Fig 2:**
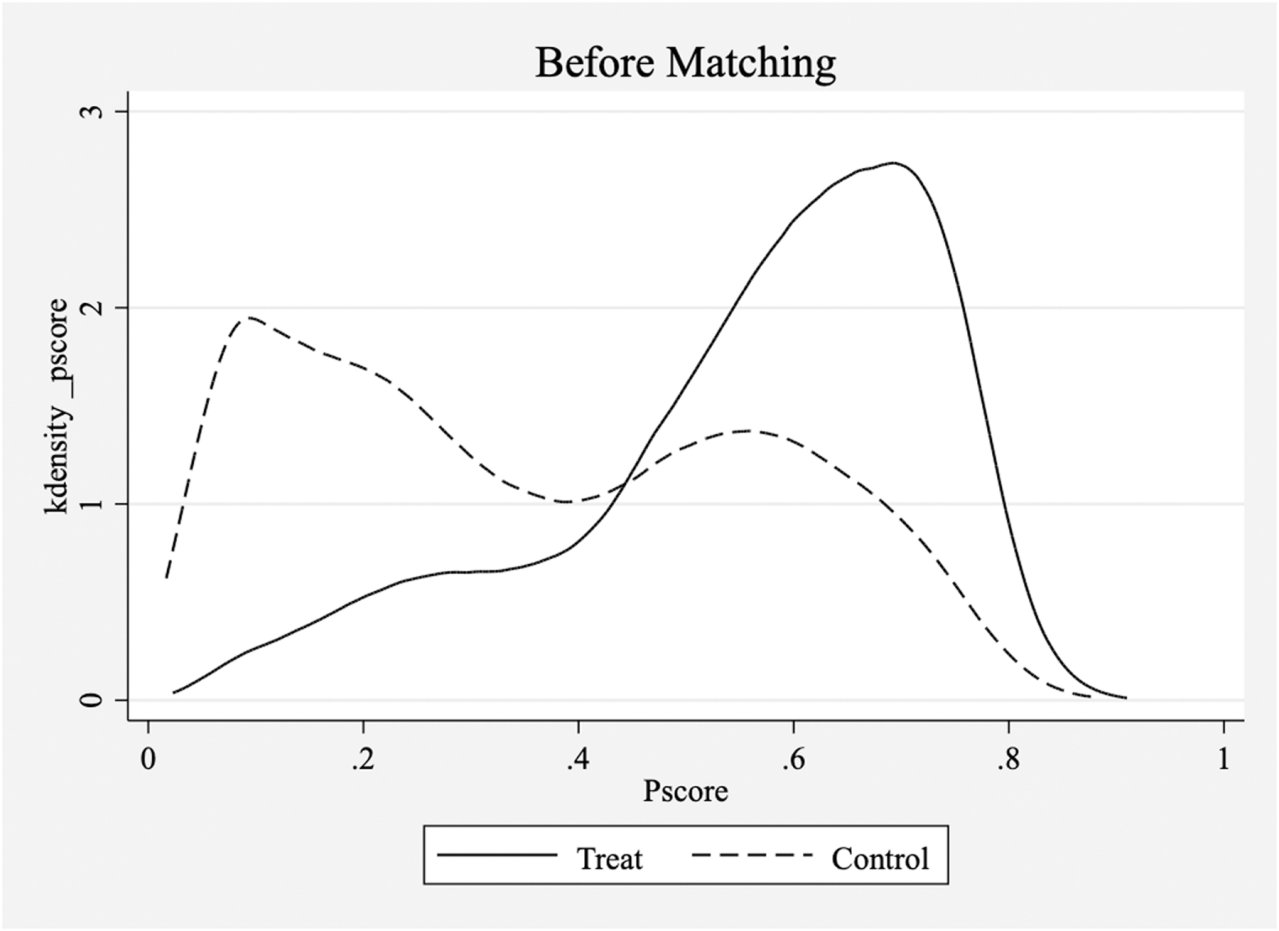
Common support process, balance test(Before).

**Fig 3:**
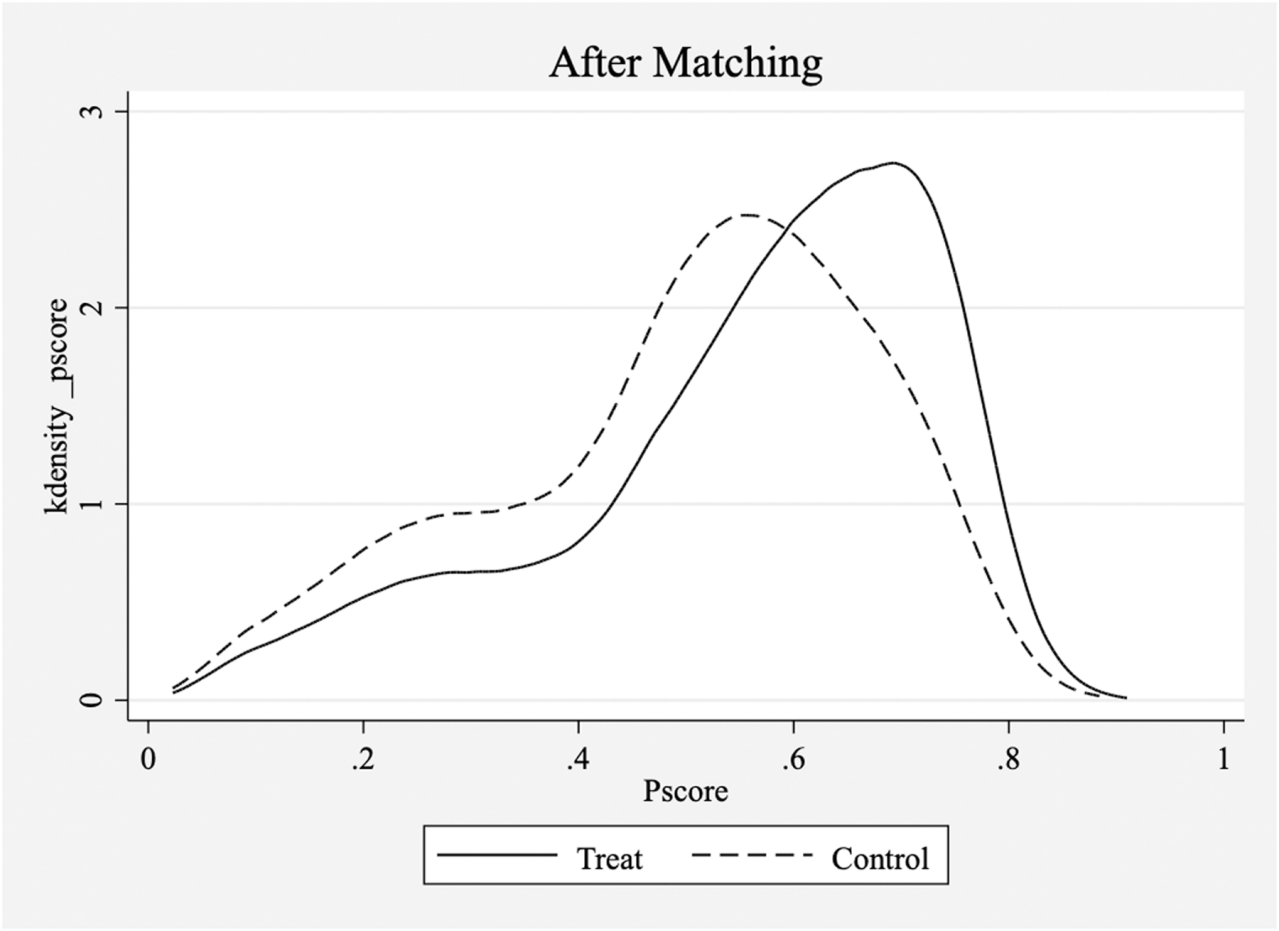
Common support process, balance test(After). 1.Notes:1. The left image shows the K-density estimation before matching, while the right image shows the K-density estimation after matching. 2.The treat group refers to listed companies that have undergone digital transformation, while the control group refers to listed companies that have not undergone digital transformation. 3.The solid line depicts the distribution of propensity scores in the treatment group, while the dashed line depicts the distribution of propensity scores in the control group.

Although we used instrumental variable (IV) methods to address endogeneity issues, it is possible that firms undergoing digital transformation differed significantly from those that did not in terms of various variables, resulting in a substantial reduction in comparability between the treatment and control groups. To address this concern, we employ propensity score matching. We treated each firm in each year as a sample unit and performing one-to-one matching without replacement between firms that underwent digital transformation and those that did not. Our aim was to ensure that the percentage difference in the propensity scores between the matched experimental and control groups was within 5%. After matching, we obtained 22,064 matched samples, representing firms from both the control and experimental groups. Subsequently, we conducted tests for common support and balance and compared kernel density plots to examine the differences in sample characteristics before and after propensity score matching to evaluate matching quality and propensity score distribution between the matched samples. The results are shown in Figs 1–3.

Figs 1–3 show the results of the common support process, balance test, and K-density estimates. The common support test and kernel density plots before matching revealed that the propensity scores for the control group were evenly distributed with two peaks, with the highest values centered on 0.1. In contrast, the experimental group showed a more concentrated propensity score distribution ranging mainly from 0.5 to 0.8, indicating significant differences between the two groups, though some overlap in the propensity score ranges is observed. However, kernel density plots after matching showed improved comparability between the treatment and control groups, with largely aligned propensity score distributions. The balance test results also demonstrated a substantial reduction in the differences between the experimental and control groups after matching, with the largest difference being <  5%, indicating a closer resemblance between the two groups. In summary, propensity score matching effectively mitigated intergroup differences in variables.

Following this approach, we matched the observable dependent variables for each listed firm over time, resulting in a set of ideal comparable experimental and control groups. The experimental group consisted of listed firms that underwent digital transformation, whereas the control group consisted of listed firms that did not undertake digital transformation. Subsequently, we conducted a fixed-effects model regression on the matched samples, and the results are presented in Table 8. Comparing the matched results with the pre-matched results, the absolute values and directions of the coefficients remain largely unchanged, providing further evidence that digital transformation by listed firms promotes the fulfillment of social responsibility and reduces the fulfillment of environmental responsibility. Specifically, Table 8 illustrates that after matching, the fixed-effects model results show that for every 1% increase in the frequency of digital transformation, the probability of a firm disclosing efforts to protect customer and consumer rights increased by 1.9%, the probability of disclosing public relations and social welfare initiatives increased by 1.4%, the probability of disclosing efforts to protect shareholder rights increased by 2.4%, and the probability of disclosing efforts to fulfill and protect employees’ social responsibilities increased by 1.6%. Moreover, the probability of disclosing environmental governance responsibilities continued to decrease. The probability of disclosing dust and smoke control efforts decreased by 1.6%, exhaust gas emission reduction control decreased by 1.4%, and wastewater emission reduction control decreased by 0.6%. However, the probabilities of disclosing wastewater emissions reduction efforts, solid waste utilization, and disposal efforts became statistically insignificant.

**Table 8:** Fixed-effects regression after PSM.

	(1)	(2)	(3)	(4)	(5)	(6)	(7)	(8)	(9)
Variable	CustomerProtection	SystemConstruction	PublicRelations	ShareholdersProtection	StaffProtection	SootDustRed_d	WasteGasEmissRed_d	WasteWaterEmissRed_d	SolidWasteDispUtil_d
Digital_transfer	0.019***	-0.007**	0.014***	0.024***	0.016***	-0.016***	-0.014***	-0.006	-0.002
	(0.004)	(0.003)	(0.004)	(0.004)	(0.004)	(0.003)	(0.004)	(0.004)	(0.004)
Control	Yes	Yes	Yes	Yes	Yes	Yes	Yes	Yes	Yes
Firm/ Year FE	Yes	Yes	Yes	Yes	Yes	Yes	Yes	Yes	Yes
Constant	-1.297***	-0.373***	-1.250***	-1.097***	-0.983***	0.091	-0.403**	-0.295 *	-0.484***
	(0.180)	(0.124)	(0.186)	(0.171)	(0.167)	(0.144)	(0.172)	(0.172)	(0.172)
Observations	22,064	22,064	22,064	22,064	22,064	22,064	22,064	22,064	22,064
R-squared	0.635	0.417	0.548	0.653	0.627	0.595	0.618	0.620	0.566

Notes:1. All regressions control for time fixed effects, individual fixed effects, and the five control variables; 2. Standard errors in parentheses. 3.The asterisks * , **, and *** indicate statistical significant at the 10%, 5%, and 1% levels.

To ensure that the treatment and control groups were comparable in terms of multiple variables and to avoid the effects of sample selection bias, we used one-to-one propensity score matching without put-back. We treated each firm as a sample unit for each year, matching firms that experienced digital transformation with those that did not and ensuring that the difference in propensity scores between the matched treatment and control groups did not exceed 5%. Using this approach, we obtained 22,064 matched samples representing firms in both the control and experimental groups. The matched data were used in subsequent fixed-effects model regression analyses to validate the impact of digital transformation on corporate social and environmental responsibility.

We then utilized propensity score matching in the IV-2SLS regression, and the conclusions obtained from Table 9 remained highly consistent with the main findings. First, the positive effect of listed firms’ digital transformation on the fulfillment of social responsibilities and the negative effect on environmental responsibilities remained unchanged. Moreover, the detailed results in the table below indicate that for every 1% increase in a firm’s digital transformation frequency, the probability of disclosing efforts to protect customer and consumer rights increased by 3.9%, the probability of disclosing efforts to protect shareholder rights increased by 7.7%, and the probability of disclosing efforts to fulfill and protect employees’ social responsibilities increased by 5.2%. All results were statistically significant at the 1% level. Specifically, the probability of disclosing environmental governance responsibilities continued to decrease. The probability of disclosing dust and smoke control efforts decreased by 11%, exhaust gas emission reduction control decreased by 8.4%, and wastewater emission reduction control decreased by 7.5%. The magnitude and direction of the coefficients are largely consistent with the results obtained from the propensity score matching.

**Table 9:** IV-2SLS regression.

	(1)	(2)	(3)	(4)	(5)	(6)	(7)	(8)	(9)
Variable	CustomerProtection	SystemConstruction	PublicRelations	ShareholdersProtection	StaffProtection	SootDustRed_d	WasteGasEmissRed_d	WasteWaterEmissRed_d	SolidWasteDispUtil_d
Digital_transfer	0.039**	-0.026**	-0.004	0.077***	0.052***	-0.110***	-0.084***	-0.075***	-0.033 *
	(0.019)	(0.013)	(0.020)	(0.018)	(0.018)	(0.016)	(0.019)	(0.019)	(0.019)
Control variable	Yes	Yes	Yes	Yes	Yes	Yes	Yes	Yes	Yes
Firm/ Year FE	Yes	Yes	Yes	Yes	Yes	Yes	Yes	Yes	Yes
Observations	22,025	22,025	22,025	22,025	22,025	22,025	22,025	22,025	22,025

Notes:1. All regressions control for time fixed effects, individual fixed effects, and the five control variables; 2. Standard errors in parentheses. 3.The asterisks * , **, and *** indicate statistical significant at the 10%, 5%, and 1% levels.

### 4.3 Reduce-form difference-in-differences (DID)

**Fig 4:**
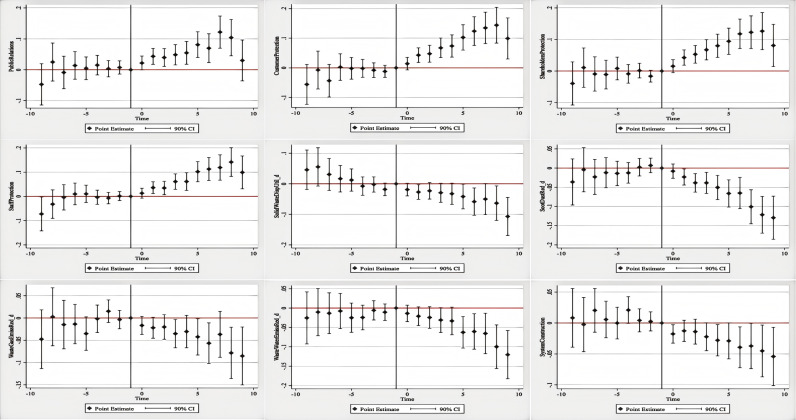
Parallel trend testing. Notes: 1. The parallel trend test results describe the changes of the coefficients and confidence intervals of the dependent variables in the reduce-form DID model before and after the digital transformation. 2. Take the year before the digital transformation of the company as the benchmark group. 3. The coefficient of the independent variables is not significantly different from 0 before the implementation of the policy but is significantly different from 0 after the implementation of the policy, which means that the parallel trend test has been passed.

In this section, we use fixed-effects models and baseline regression to investigate the influence of digital transformation on social and environmental disclosures using the natural logarithm of a firm’s digital transformation frequency as an independent variable. However, if a firm consistently discloses terms related to digital transformation, it should be regarded as either undergoing digital transformation or having a propensity for it. We define a firm’s digital transformation as follows: if a firm discloses relevant terms related to big data, cloud computing, blockchain, digital technology applications, and artificial intelligence in a particular year and onwards, we consider the firm to have implemented digital transformation and assign a value of 1 to this indicator; otherwise, we assign a value of 0 to the indicator. Propensity score matching is based on this classification, matching firms with a value of 1 (indicating digital transformation) to firms with a value of 0 (indicating no digital transformation), resulting in comparable experimental and control groups. Building on this, we further explore the dynamic effects of digital transformation on corporate social responsibility and environmental responsibility disclosures using the reduced-form DID model. The coefficients and their confidence intervals reflecting the pre- and post-digital transformation periods in the reduced-form DID model are presented in the figure below to illustrate the dynamic effects of digital transformation on environmental and social responsibility disclosures. We use the year before a firm implemented digital transformation as the reference group, and the time trend is shown in Fig 4.

From Fig 4, we can see that not all dependent variables are significantly different from zero in the ten years before digital transformation, validating the parallel trends assumption of the difference-in-differences approach. This assumption indicates that there should be no systematic differences in the dependent variables between the experimental and control groups before and after the digital transformation. Subsequently, we observed that four variables: “Disclosure of public relations and social welfare initiatives,” “Disclosure of efforts to protect customer and consumer rights,” “Disclosure of efforts to protect shareholder rights,” and “Disclosure of efforts to protect employee rights” all exhibited promotion effects one year after the policy change without any lag effects. However, the variable “Disclosure of efforts to construct and improve social responsibility systems” shows some lag effects, with its impact being less pronounced. The effect of digital transformation on environmental disclosures shows some lag effects, indicating that digital transformation inhibited environmental protection disclosures within 2–5 years of policy change. These results are consistent with the baseline regression results, demonstrating the robustness of our conclusions.

### 4.4 Robustness checks

We also conducted a series of robustness checks to demonstrate the reliability of our findings. In Table 10, we exclude five control variables from the baseline regression: the firm’s total assets, age, price-to-book ratio, debt-to-assets ratio, and ownership percentage of the largest shareholder. Following propensity score matching, we proceeded with the second-stage regression for which the first-stage regression results were previously reported. Our analysis revealed that the conclusions of the baseline regression are consistent. The likelihood of firms disclosing efforts to protect customer and consumer rights, disclosing efforts to protect shareholder rights, and disclosing efforts to fulfill and protect employee social responsibilities continues to exhibit a positive association with the advancement of digital transformation, and these results remain statistically significant at the 1% level. Conversely, the probabilities of firms disclosing dust and smoke control efforts, exhaust gas emission reduction control efforts, and wastewater emission reduction control efforts are negatively affected by digital transformation. The absolute values and directions of the coefficients largely align with the results obtained from the propensity score matching, signifying minimal influence on the main regression model when controlling for firm and market variables.

**Table 10:** Robustness checks(a).

	(1)	(2)	(3)	(5)	(6)	(7)	(8)	(9)	(10)
Variable	CustomerProtection	SystemConstruction	PublicRelations	ShareholdersProtection	StaffProtection	SootDustRed_d	WasteGasEmissRed_d	WasteWaterEmissRed_d	SolidWasteDispUtil_d
Digital_transfer	0.055***	-0.023 *	0.015	0.089***	0.065***	-0.102***	-0.072***	-0.068***	-0.025
	(0.020)	(0.013)	(0.019)	(0.017)	(0.017)	(0.015)	(0.017)	(0.017)	(0.017)
Control variable	No	No	No	No	No	No	No	No	No
Firm/ Year FE	Yes	Yes	Yes	Yes	Yes	Yes	Yes	Yes	Yes
Observations	22,025	22,025	22,025	22,025	22,025	22,025	22,025	22,025	22,025

Notes:1. All regressions control for time fixed effects, individual fixed effects; 2. Standard errors in parentheses. 3.The asterisks * , **, and *** indicate statistical significant at the 10%, 5%, and 1% levels.

Additionally, we incorporate province and city fixed effects into the regression alongside time and firm fixed effects while simultaneously controlling for the same five control variables affecting firm behavior. After propensity score matching, we proceed with the IV-2SLS two-stage regression, and the results are shown in Table 10. The outcomes in Table 11 demonstrate that the coefficient magnitudes, directions, and economic implications remain similar to those obtained without considering the province and city fixed effects. This reaffirms the assertion that digital transformation by listed firms fosters the fulfillment of social responsibilities and reduces the fulfillment of environmental responsibilities, thereby reinforcing the robustness of our conclusions.

**Table 11:** Robustness checks(b).

	(1)	(2)	(3)	(4)	(5)	(6)	(7)	(8)	(9)
Variable	CustomerProtection	SystemConstruction	PublicRelations	ShareholdersProtection	StaffProtection	SootDustRed_d	WasteGasEmissRed_d	WasteWaterEmissRed_d	SolidWasteDispUtil_d
Digital_transfer	0.042**	-0.028**	-0.009	0.081***	0.056***	-0.110***	-0.090***	-0.076***	-0.035 *
	(0.020)	(0.014)	(0.020)	(0.019)	(0.018)	(0.016)	(0.019)	(0.019)	(0.019)
Control variable	Yes	Yes	Yes	Yes	Yes	Yes	Yes	Yes	Yes
Prov/ City/ Firm/ Year FE	Yes	Yes	Yes	Yes	Yes	Yes	Yes	Yes	Yes
Observations	22,025	22,025	22,025	22,025	22,025	22,025	22,025	22,025	22,025

Notes:1. All regressions control for firm fixed effects, year fixed effects, province and city fixed effects, the five control variables; 2. Standard errors in parentheses. 3.The asterisks * , **, and *** indicate statistically significant at the 10%, 5%, and 1% levels.

### 4.5 Mechanism

We used fixed effects, an instrumental variables method, and a simplified double difference method and found a positive relationship between firms’ digital transformation and disclosure of CSR fulfilment as well as a negative relationship between digital transformation and CSR fulfilment and addressed the endogeneity issue with propensity score matching. Notably, enterprise digital transformation has a facilitating effect on the social responsibility of stakeholders, such as employees, consumers, and stockholders of the enterprise, and presents a dampening effect on the disclosure of environmental responsibility. The rationale behind these dynamics is intuitive. Digital transformation can reduce information transmission costs among employees and enhance transparency and equality among them, consequently promoting the fulfillment of employee responsibilities. This positive effect can be considered promotional. Conversely, in terms of the relationship between digital transformation and environmental responsibility, the higher computational power and energy consumption demands of digital transformation may lead to increased carbon emissions and additional environmental pollution due to the solid and gaseous waste generated by the equipment. Furthermore, both digital transformation and environmental responsibility require resource-intensive processes, entailing significant financial support and resource constraints, whereas social responsibility fulfillment is more human-centric and does not require significant material resources.

Consequently, financing constraints may influence the competitive relationship between digital transformation and environmental responsibility. Prior research proposes the KZ index as a useful metric for assessing a firm’s financing constraints, with higher KZ index values indicating more substantial financing constraints faced by a firm. Building on the literature [18], we incorporate the KZ index into the analysis of mediating effects to gain insight into its influence. The results are presented in Table 12. The findings indicate that while digital transformation levels remain unchanged, higher financing constraints lead to reduced environmental responsibility fulfillment by firms, suggesting an exclusive competitive effect between digital transformation and environmental responsibility. Surprisingly, higher financing constraints promote the fulfillment of CSR obligations to stakeholders, suggesting that financing constraints act as an incentive for social responsibility fulfillment.

**Table 12:** Mediating Effect of Financing Constraints on Digital Transformation and Responsibility Fulfillment.

	(1)	(2)	(3)	(4)	(5)	(6)	(7)	(8)	(9)
Variable	CustomerProtection	SystemConstruction	PublicRelations	ShareholdersProtection	StaffProtection	SootDustRed_d	WasteGasEmissRed_d	WasteWaterEmissRed_d	SolidWasteDispUtil_d
KZ index	0.002**	-0.000	0.002***	0.002***	0.002***	-0.002***	-0.002***	-0.002***	-0.002***
	(0.001)	(0.000)	(0.001)	(0.001)	(0.001)	(0.001)	(0.001)	(0.001)	(0.001)
Digital_transfer	0.014***	-0.001	0.003	0.014***	0.007**	-0.017***	-0.008***	-0.003	-0.001
	(0.003)	(0.002)	(0.003)	(0.003)	(0.003)	(0.003)	(0.003)	(0.003)	(0.003)
Control variable	Yes	Yes	Yes	Yes	Yes	Yes	Yes	Yes	Yes
Constant	-1.284***	-0.527***	-1.202***	-1.132***	-1.041***	-0.015	-0.491***	-0.442***	-0.786***
	(0.135)	(0.095)	(0.137)	(0.129)	(0.127)	(0.112)	(0.130)	(0.131)	(0.129)
Firm/ Year FE	Yes	Yes	Yes	Yes	Yes	Yes	Yes	Yes	Yes
Observations	31,564	31,564	31,564	31,564	31,564	31,564	31,564	31,564	31,564
R-squared	0.595	0.383	0.536	0.616	0.597	0.524	0.558	0.563	0.507

Notes:1. All regressions control for firm fixed effects, year fixed effects, and the five control variables; 2. Standard errors in parentheses. 3.The asterisks * , **, and *** indicate statistical significant at the 10%, 5%, and 1% levels.

### 4.6 Heterogeneity analysis

**Fig 5:**
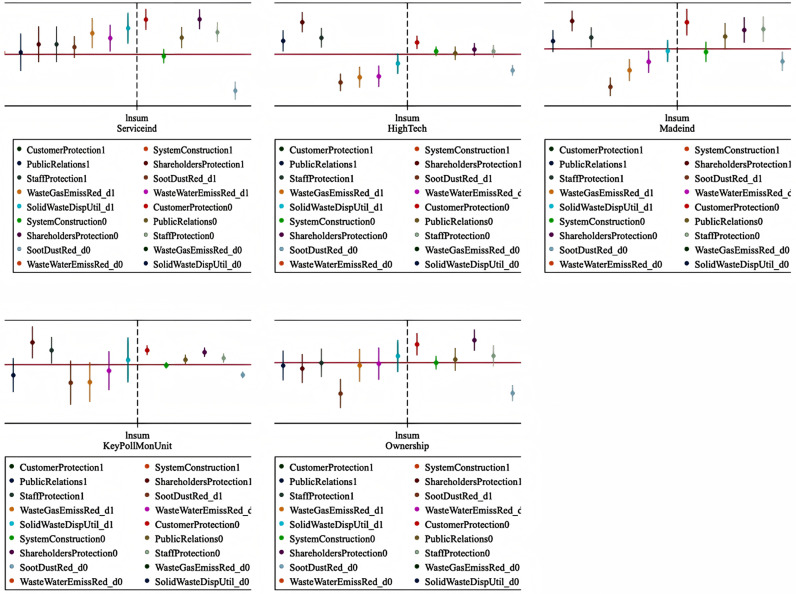
Heterogeneity analysis. Notes: 1. The above figure conducts heterogeneity analysis on whether the company is a manufacturing, high-tech, state-owned, heavily polluting, or manufacturing enterprise. 2. On the left side of the vertical dashed line are heterogeneity variables defined as 1, and on the right side are heterogeneity variables defined as 0. 3. Above the horizontal zero baseline means that the digital transformation has a positive impact on this heterogeneous variable, and below it means that the sigital transformation has a negative impact on this heterogeneous variable.

This section presents a heterogeneity analysis of the impacts on different groups of firms. Generally, firms of different natures, societies, and institutions have different requirements for CSR fulfillment. Society and institutions expect state-owned enterprises to have a higher degree of social responsibility and environmental protection than non-state-owned enterprises. Moreover, owing to the differing industrial nature of enterprises, the emphasis on employees, customers, and shareholders during the digital transformation process and the emphasis on environmental responsibility may vary. For example, manufacturing enterprises may focus on fulfilling their environmental responsibilities to ensure that the production process complies with institutional norms and products meet market demands. Service enterprises may pay more attention to the rights and interests of employees and consumers while neglecting the disclosure of environmental responsibilities. High-tech enterprises may have more means of solving environmental problems; however, because most high-tech enterprises are based in the manufacturing industry, their emphasis on employees, customers, and shareholders may not be high. Heavily polluting enterprises that are widely concerned with society as its focus not only pay more attention to their own environmental responsibilities for self-improvement but also pay more attention to their reputation, with an emphasis on employees, customers, and shareholders. Therefore, we conduct a heterogeneity analysis on manufacturing enterprises, high-tech enterprises, state-owned enterprises, manufacturing industries, and heavily polluting companies. The results are shown in Fig 5. We found that the impact of digital transformation on service enterprises is positive in terms of the direction of impact, and there is no negative impact on the nine indicators. Furthermore, the disclosure of environmental responsibilities has a positive impact, indicating that the digital transformation of service enterprises corresponds to better social responsibility performance. High-tech enterprises are more likely to disclose the construction and improvement measures of social responsibility systems, public relations and social welfare, shareholder rights protection, and employee rights protection, while the probability of disclosing related environmental responsibilities is relatively low. Overall, high-tech enterprises respond more dramatically to digital transformation. The performance of manufacturing enterprises in the disclosure of environmental and social responsibilities and the protection of stakeholders’ rights and interests is consistent with the overall sample. For heavily polluting enterprises, the impact of their digital transformation on the disclosure of environmental responsibilities is not significant and differs from the repulsion between digital transformation and environmental responsibilities shown by other enterprises. Notably, we find a significant difference between state-owned and non-state-owned enterprises in the fulfillment of social and environmental responsibilities. The main impact of digital transformation is on non-state-owned enterprises, whereas the disclosure of ESG responsibility of state-owned enterprises is not affected by the digital transformation process. These results contradict our hypothesis, possibly because the adjustment speed of state-owned enterprises affected by policies is slower. In general, firms are affected differently by digital transformation.

## 5. Discussion

### 5.1 Conclusion

Based on data from China’s A-share listed companies from 2008 to 2021, this study explored the impact of enterprise digital transformation on corporate social and environmental responsibility using Python technology to construct enterprise digital transformation indicators. Based on the empirical study, the following conclusions are drawn. First, it is found that enterprise digitalization affects corporate social and environmental responsibility in diametrically opposite directions. Enterprise digital transformation has a positive impact on corporate social responsibility, while it has a negative impact on environmental responsibility. Second, financing constraints present a positive moderating effect between enterprise digital transformation and CSR and a negative moderating effect between corporate environmental responsibility. Finally, digital transformation has different effects on CSR and environmental responsibility for different types of enterprises.

### 5.2 Research contribution

Compared with existing studies, the main contributions of this study are as follows: First, by taking Chinese A-share listed companies as the research sample, we comprehensively examine the impact of digital transformation on enterprise social and environmental responsibility, extend existing studies, and provide theoretical support for the fulfilment of the dual responsibility of society and the environment in the process of enterprise digital transformation. Second, this paper contributes to the context of corporate social and environmental responsibility fulfilment by selecting the internal factor of corporate financing constraints as the moderating context. Doing so enriches the contextual examination under the basic regression and deepens the effect of the influence of different contexts on social and environmental responsibility in the process of corporate digital transformation. Finally, this study introduces the micro characteristics of enterprises and the characteristics of the industries to which they belong (such as property rights, high-tech level, and industry category) into an empirical analysis to explore the heterogeneous effects of digital transformation on enterprises’ social and environmental responsibility. This provides an important reference for enterprises to achieve digital transformation and low-carbon green development.

### 5.3 Practical insight

Insight was gained through the research in this paper. First, enterprises should attach great importance to the social and environmental spillover effects of digital transformation. Additionally, while integrating their daily business activities into the digital transformation strategy, they should also shoulder their social and environmental responsibilities to achieve value creation in the non-economic performance of enterprise digital transformation. Second, enterprises should be encouraged to build a social and environmental responsibility governance system based on digital technology and oriented toward responsibility and value, leading them to be more proactive in assuming social and environmental responsibility in the process of enterprise digital transformation and achieving sustainable economic development.

### 5.4 Limitations and further research

The research may have some limitations: firstly, this paper chooses word frequency as a measure of digital transformation and selects keywords more subjectively. As each researcher has different subjective criteria and different selection ranges, the keywords chosen by different articles vary, which makes the digital transformation indicators based on word frequency lack comparability. Second, digital technology is constantly updated and new terms are constantly emerging, so using the word frequency method to measure digital transformation will inevitably result in a slow update of word frequency, which is difficult to reflect the latest vocabulary of digital transformation, so the use of the word frequency method has a certain omission problem. Finally, although the instrumental variable method as well as the propensity score method are used to mitigate the endogeneity problem during the empirical process of this paper, it still does not solve all possible endogeneity problems.

This paper explores the application of digital transformation in enterprises from a new perspective, but the study is limited to the impact on corporate social and environmental responsibility. In the future, we can focus on the following research work: first, due to certain shortcomings of the word frequency method, we should continue to search for a measurement method that can more accurately reflect digital transformation in the future; second, in the future, we can further delve into the impact of digital transformation on corporate social and environmental responsibility, such as from the perspective of source control or end-of-pipe governance, to further refine the impact of digital transformation on corporate social and environmental responsibility.

## Supporting information

S1 Data Dataset. (XLS)
